# Pembrolizumab-Induced Myasthenia Gravis and Peripheral Neuropathy: A Case Series

**DOI:** 10.7759/cureus.44799

**Published:** 2023-09-06

**Authors:** Sean M McCormack, Amar Hamad

**Affiliations:** 1 Medicine, Saint James School of Medicine, Park Ridge, USA; 2 Hematology and Medical Oncology, Affiliated Oncologists, Chicago Ridge, USA

**Keywords:** pembrolizumab-induced peripheral neuropathy, pembrolizumab-induced myasthenia gravis, immune checkpoint inhibitor (ici), programmed death 1 (pd-1), oncology case report, pembrolizumab, cancer immunotherapy, programmed death ligand 1 (pd-l1), immune-related adverse events (iraes), pembrolizumab side effect

## Abstract

Pembrolizumab is a monoclonal antibody that targets the programmed cell death protein 1 (PD-1) receptor on T-cells, thereby enhancing the antitumor immune response. Pembrolizumab has been shown to improve survival in various cancers, but it can also cause immune-related adverse events (irAEs), which can affect any organ system. We report two cases of rare but serious irAEs caused by pembrolizumab: myasthenia gravis (MG) and peripheral neuropathy. Both patients presented with neuromuscular symptoms after receiving pembrolizumab for their advanced cancers. They were diagnosed with MG and peripheral neuropathy based on their clinical features, laboratory tests, and unremarkable imaging. Treatment involved discontinuing pembrolizumab and initiating immunosuppressive and supportive therapies. Both patients experienced improvement in their symptoms and quality of life once pembrolizumab was permanently discontinued and supportive therapies were in place. These cases highlight the importance of recognizing and managing rare irAEs of pembrolizumab, such as MG and peripheral neuropathy. Early diagnosis and treatment can improve outcomes and reduce morbidity. Furthermore, these cases emphasize the need for continued post-marketing surveillance to accurately assess the risk of less frequent adverse drug reactions seen in patients on pembrolizumab. Knowledge of these adverse reactions is important when discussing the pros and cons of this novel therapy with patients.

## Introduction

Immune checkpoint inhibitors (ICIs) can be divided into three general categories: programmed cell death protein 1 (PD-1) inhibitors (cemiplimab, nivolumab, and pembrolizumab), programmed death ligand 1 (PD-L1) inhibitors (atezolizumab, avelumab, and durvalumab), and cytotoxic T-lymphocyte antigen 4 (CTLA-4) inhibitor (ipilimumab). All of these ICIs have been approved by the Food and Drug Administration (FDA) for the treatment of various types of cancer.

Pembrolizumab is a novel monoclonal antibody that has revolutionized the treatment of various cancers, including melanoma, cutaneous squamous cell carcinoma, triple-negative breast cancer, and non-small cell lung cancer, among others. Pembrolizumab was initially approved by the FDA on September 4, 2014, for the treatment of patients with unresectable or metastatic melanoma. Since then, the FDA indication for pembrolizumab has been expanded to many different cancers at various stages [1,2]. Pembrolizumab has been shown to improve both overall survival and progression-free survival in patients with many different cancers. Pembrolizumab works by blocking the interaction between programmed cell death protein 1 (PD-1) and its ligands, programmed death ligand 1 (PD-L1) and programmed death ligand 2 (PD-L2), thus enhancing the antitumor immune response [3]. However, like all drugs, pembrolizumab has side effects, which can range from mild to severe. The most common side effects of pembrolizumab include fatigue, rash, pruritus, diarrhea, nausea, and arthralgia. These side effects are usually mild to moderate in severity and can be managed with supportive care or dose modifications. However, pembrolizumab can also cause immune-related adverse events (irAEs). While irAEs were once thought to be very rare, with the widespread adoption of ICIs such as pembrolizumab, we are seeing more people experiencing the adverse effects of these drugs. One of the rarer side effects of pembrolizumab is the development of myasthenia gravis (MG), an autoimmune neuromuscular disease. Another rare side effect of pembrolizumab is peripheral neuropathy, which is characterized by tingling, numbness, and/or pain in the hands and feet [4-6]. In this case series, first, we present a patient who developed myasthenia gravis as a side effect of pembrolizumab. Then, we present a patient who developed peripheral neuropathy secondary to pembrolizumab. It is important that these rare neurological side effects are reported. Through this case series, we aim to bring awareness of these less common side effects of pembrolizumab to the medical community. It is important for patients to be aware of these potential side effects as rare as they may be. Establishing data to accurately present the risk of adverse drug reactions is important when discussing the pros and cons of these novel medications with patients.

## Case presentation

Case 1

A 74-year-old female with a history of stage II left upper outer quadrant triple-negative breast cancer had received neoadjuvant chemotherapy prior to lumpectomy with wide excision, adjuvant radiation therapy, and one cycle of adjuvant immunotherapy with pembrolizumab. Her past medical history included hypertension, end-stage renal disease on dialysis, chronic systolic heart failure, stroke, malnutrition, and rheumatoid arthritis. The patient denied any family history of diseases. Her medications included acetaminophen, hydroxychloroquine, gabapentin, amlodipine, guaifenesin, famotidine, valsartan, isosorbide dinitrate, prochlorperazine, polyethylene glycol, docusate sodium, ondansetron, clonidine, furosemide, hydralazine, carvedilol, febuxostat, and a multivitamin.

She presented to the hospital one week after starting her first session of immunotherapy with pembrolizumab. The patient had present symptoms of severe generalized weakness and difficulty walking. The patient reported falling out of bed due to weakness and that this was not her baseline. On examination, she had normal vision and weakness in her neck flexors and proximal limb muscles. Her deep tendon reflexes were grossly intact, and she had no sensory deficits, no rash, and normal post-surgical changes on her left breast. The rest of her physical examination was unremarkable.

Computed tomography (CT) and magnetic resonance imaging (MRI) of the head and spine showed no acute findings. Creatine kinase and aldolase were within normal limits. All other laboratory findings were insignificant or non-contributory. She was treated for suspected immune checkpoint inhibitor (ICI)-related myositis. The patient received intravenous (IV) solumedrol Q8 hours during hospitalization and was switched to oral prednisone 50 mg daily upon discharge. Her symptoms gradually improved, and she was discharged from the hospital after a week. The patient reported strong improvement of extremity weakness at follow-up one week after discharge. The patient continued to improve with physical therapy exercises at home. The patient was able to ambulate well with the assistance of a cane. Prednisone was tapered over the course of two months down to 10 mg daily.

The patient began to experience a gradual worsening of extremity weakness and difficulty walking while on the lower dose of prednisone. This ultimately led to hospital readmission due to an increase in symptoms. Repeat CT and MRI of the head and spine showed no acute findings. The patient received electromyography (EMG), and the test showed abnormal repetitive nerve stimulation and severe axonal polyneuropathy in the lower extremities. These findings are consistent with neuromuscular junction disorders such as myasthenia gravis. The patient was tested for acetylcholine receptor antibodies and was negative. Based on her clinical presentation, examination findings, and test results, the patient was diagnosed with myasthenia gravis. Prednisone was increased to 50 mg daily, and the patient received plasmapheresis daily for five days. At discharge, the patient was feeling significantly better. Again, at her follow-up after discharge, the patient was able to ambulate without assistance. Over the course of several months, prednisone was tapered and stopped. Again, the patient began to experience increased muscle weakness without steroids. She began to develop significant dysmotility in the esophagus and stomach. These worsening symptoms were concerning and thought to be associated with her diagnosis of myasthenia gravis. Due to the recurrence of symptoms whenever prednisone was tapered or discontinued, it was hypothesized that her condition is cortisol-dependent. Prednisone was restarted indefinitely to maintain remission of symptoms. An appropriate dose was determined to balance the risks and benefits of long-term steroid use. The patient began treatment with prednisone 20 mg daily with improvement of symptoms. The patient has continued to receive maintenance prednisone at 20 mg daily for multiple months. She will likely continue on prednisone therapy in the long term as her disease state seems to be cortisol-dependent. While the patient continues to receive alternative treatment for her breast cancer, she has not been restarted on pembrolizumab.

Case 2

A 71-year-old male with a history of stage III squamous cell carcinoma of the skin has been receiving pembrolizumab as part of his treatment plan. He has had innumerable skin lesions treated in the past, ranging from actinic keratosis to melanoma in situ to squamous cell carcinoma with positive margins. Due to the sheer amount of lesions and frequent recurrence, he was started on pembrolizumab 1.5 years ago, which he had been tolerating well with no reported side effects, except for a short episode of immune colitis that was successfully treated with oral prednisone. The patient has a significant past medical history, including hypertension, well-controlled diabetes mellitus, weight loss, and myocardial infarction with stent placement. He has also undergone various surgical procedures, including orthopedic surgeries, carpal tunnel surgery, and implantation of a cardiac pacemaker, as well as numerous skin resections before and during pembrolizumab treatment. At a typical visit with the dermatologist, it was common for the patient to have 20-30 lesions treated with surgical excision, thermal ablation, and topical 5-fluorouracil. The patient’s history is also positive for a latex allergy, and socially, he is a former smoker.

In the two weeks leading up to him presenting to the clinic, the patient’s symptoms of peripheral neuropathy both developed and worsened acutely. At his presentation, he was experiencing severe, debilitating, painful numbness and tingling in his hands and arms bilaterally. This started as minor tingling in his fingers and gradually progressed to severe numbness and pain over several weeks. The tingling was constant, and there were no relieving factors. He had weakness in his hands, along with difficulty performing fine motor tasks. On physical examination, the patient had subjective decreased sensation to light touch and pinprick in a stocking-glove distribution, with slight weakness and atrophy noted in interossei hand muscles. Arm muscle strength and tone were normal bilaterally. The patient however reported some generalized weakness, which he attributed to his weight loss over the past year. Notably, the patient had not received systemic chemotherapy or other medical therapies known to frequently cause peripheral neuropathy, and his diabetes remained well controlled. Despite this, he has developed signs and symptoms suggestive of peripheral neuropathy with no changes in his treatment and no other disease progression. The patient was subsequently tested for acetylcholine receptor antibodies, creatine kinase, and C-reactive protein, all of which were normal. An MRI of the head and cervical spine without contrast was performed and showed no acute findings. The MRI showed mild brain atrophy appropriate for age and moderate spinal stenosis at C3-C4 without spinal cord lesions or significant compressions. Electromyography (EMG) was performed on both upper extremities, and the findings were suggestive of an inflammatory demyelinating polyneuropathy. It was ultimately determined that this was likely secondary to pembrolizumab use. As a result, pembrolizumab was discontinued, and the patient was treated with prednisone. After discontinuing pembrolizumab and the completion of the prednisone taper, the patient noted a substantial improvement. There was nearly a complete improvement in symptoms over the first week, and within a month, he was asymptomatic. This further confirmed our belief that the neuropathy was a side effect of pembrolizumab use. The patient was subsequently switched to a new treatment regimen due to the side effects he experienced on pembrolizumab. The patient continues to be asymptomatic in terms of signs and symptoms of any sort of peripheral neuropathy.

## Discussion

Pembrolizumab is a monoclonal antibody that targets the programmed cell death protein 1 (PD-1) receptor on T-cells, thereby blocking the interaction between PD-1 and its ligands, PD-L1 and PD-L2. This leads to the activation of T-cells, which can then recognize and attack cancer cells. Pembrolizumab has been shown to improve overall survival and progression-free survival in various cancers, including but not limited to melanoma, cutaneous squamous cell carcinoma, triple-negative breast cancer, and non-small cell lung cancer [1].

The most common side effects of pembrolizumab include rash, pruritus, pneumonitis, colitis, hypothyroidism, nausea, and arthralgia. Other symptoms often associated with drugs targeting PD-1 are fatigue and diarrhea. However, studies have shown that these symptoms are not significantly increased compared to controls. These side effects are usually mild to moderate in severity and can be managed with supportive care or dose modifications. Some of these side effects can be classified as immune-related adverse events (irAEs), which are caused by the activation of T-cells against normal tissues. irAEs can affect any organ system, and their severity can range from mild to life-threatening. Common irAEs include pneumonitis, colitis, hepatitis, endocrinopathies, and dermatitis [4,7]. The incidence of irAEs with pembrolizumab is higher than with other immune checkpoint inhibitors, possibly due to its higher affinity for PD-1 [8].

Pembrolizumab-induced MG

Pembrolizumab-induced MG is a rare but potentially severe irAE that can occur in patients undergoing treatment with pembrolizumab. The incidence of pembrolizumab-induced MG is unknown, and estimates have significant variance. With the increasing use of pembrolizumab, we are seeing more cases reported in the literature. In a recent review of pembrolizumab-induced MG cases, it was found that the median time to onset of symptoms was four weeks. However, the range of onset is much more broad. Clinicians should consider the possibility of pembrolizumab-induced MG, especially during the initial weeks of therapy. A high level of suspicion can lead to faster interventions. All patients should be treated as early as possible, regardless of the severity of symptoms [9].

MG is an autoimmune disorder that is caused by the production of autoantibodies against acetylcholine receptors or other components of the neuromuscular junction. However, there is no consistent association between elevated acetylcholine antibody titers and the development of ICI-related MG. Seronegative MG is commonly seen secondary to ICI use [10]. The symptoms of MG can vary widely but typically include muscle weakness, particularly in the ocular, bulbar, and proximal limb muscles. This causes ptosis, diplopia, and fatigability. The diagnosis of MG can be confirmed by the presence of autoantibodies, abnormal electromyography findings, and response to cholinesterase inhibitors or immunosuppressive therapy. MG is commonly associated with thymoma. Reviewing past CT images for the presence of thymoma is helpful, as this can rule out MG-associated thymoma versus other causes [9].

The mechanism by which pembrolizumab induces MG is unknown. It has been suggested that pembrolizumab may trigger an autoimmune response by activating autoreactive T-cells or inhibiting regulatory T-cells against the neuromuscular junction. Another proposed mechanism is the enhancement of antibody-dependent cell-mediated cytotoxicity (ADCC), which may lead to the destruction of acetylcholine receptors. Additionally, other immune cells may play a role in the development of irAEs. B-cells are thought to secrete antibodies that may contribute to toxicity. A negative test for acetylcholine receptor antibodies cannot completely rule out MG as seronegative MG does exist [11].

The diagnosis of pembrolizumab-induced myasthenia gravis is based on the patient’s clinical presentation and examination findings, as well as the exclusion of other causes of neuromuscular weakness. Treatment involves the discontinuation of pembrolizumab and the initiation of immunosuppressive therapy, such as corticosteroids, intravenous immunoglobulin (IVIG), and plasmapheresis. Immunosuppressive therapy may be given in combination, especially in cases that are severe or unresponsive to treatment. The prognosis of pembrolizumab-induced myasthenia gravis is generally good, with most patients experiencing improvement in their symptoms after the discontinuation of pembrolizumab and the initiation of immunosuppressive therapy [9,12]. Until symptoms are in remission, it is important to monitor for respiratory depression as it can have the most severe consequences on mortality. The mortality rate of ICI-mediated MG is 30% [10].

It is important to note that in this case, the patient was taking hydroxychloroquine for rheumatoid arthritis. Hydroxychloroquine is an immunomodulatory drug used in rheumatoid arthritis with several mechanisms of action. Hydroxychloroquine and chloroquine are weak bases that accumulate in acidic organelles such as lysosomes and endosomes. They are thought to interfere with antigen processing and presentation by inhibiting lysosomal proteases. They also inhibit Toll-like receptor (TLR) signaling by blocking endosomal acidification. In addition, they have been shown to inhibit autophagy. Hydroxychloroquine can also be useful in reducing the production of pro-inflammatory cytokines. These mechanisms of action have implications for rheumatology and other autoimmune diseases [13]. Hydroxychloroquine has been shown to induce MG on its own, as has pembrolizumab [7,14]. While we do not believe that the patient’s MG was triggered by hydroxychloroquine alone, as she had been taking this for the long term, it could be that an interaction between the immunomodulatory effects of both hydroxychloroquine and pembrolizumab occurs. A possible drug-drug interaction may have a synergistic effect on the development of MG when she was started on pembrolizumab. However, more research is needed to test this hypothesis.

Pembrolizumab-induced neuropathy

Pembrolizumab-induced neuropathy is a rare but recognized side effect of the drug. While it does occur, the rate at which it is seen is less than placebo and less than traditional chemotherapy regimens [15]. The exact mechanism of how pembrolizumab causes neuropathy is not well understood, but it is thought to be related to its immune-enhancing properties. The immune system can attack the peripheral nerves, leading to inflammation and damage. T-cells are believed to play an important role in ICI-mediated toxicity. However, due to a wide variety of irAEs, other potential immune cells and/or cytokines likely drive these adverse reactions with multiple mechanisms of action [16]. The onset of neuropathy can vary from several weeks to several months after starting pembrolizumab but can occur at any time during treatment [17].

The differential diagnosis for peripheral neuropathy includes a wide range of conditions, such as diabetes mellitus, alcoholism, vitamin deficiencies, autoimmune disorders, infections, and exposure to toxins. In this case, the patient had no history of diabetes or alcohol abuse, and laboratory tests ruled out vitamin deficiencies and infections. A nerve conduction study and electromyography (EMG) can be performed. Evidence of axonal neuropathy with reduced sensory and motor amplitudes is consistent with a diagnosis of peripheral neuropathy [18].

It is important to consider other causes of neuropathy in patients receiving cancer treatment, as chemotherapy and radiation therapy can also cause peripheral neuropathy. In this case, the patient had not received chemotherapeutic agents, which are known to cause neuropathy before starting pembrolizumab. The lack of other chemotherapeutic agents in his treatment along with the timing of the resolution of symptoms is more consistent with pembrolizumab-induced neuropathy than other causes such as chemotherapy [15]. It was also important to rule out diabetes mellitus as the cause of neuropathy. The neuropathy observed in this patient presented acutely and went into remission after both steroid use and discontinuing pembrolizumab. This supports pembrolizumab-induced neuropathy versus diabetes mellitus. Additionally, the pattern of nerve damage on EMG further supported the diagnosis of pembrolizumab-induced neuropathy [19,20]. Similar to other irAEs seen with ICI treatment involves discontinuing the offending drug and initiation of steroids if symptoms persist. Additionally, patients experiencing peripheral neuropathy can be offered gabapentin, pregabalin, or duloxetine for pain [12].

## Conclusions

Pembrolizumab is a monoclonal antibody that has revolutionized the treatment of various cancers. However, like all drugs, pembrolizumab has side effects, which can range from mild to severe. Pembrolizumab-induced MG is a rare but potentially severe irAE that can occur in patients undergoing treatment with pembrolizumab. The incidence of pembrolizumab-induced MG is unknown, and estimates vary significantly. Pembrolizumab-induced neuropathy is another rare but potentially debilitating side effect of the drug. Clinicians should be aware of these rare side effects and consider them in patients who present with muscle weakness, ptosis, and diplopia, as well as distal extremity tingling with progression to numbness and pain while on pembrolizumab. Early recognition and discontinuation of the drug can improve outcomes. While we are unsure of the exact mechanism that leads to these neurological adverse events, we do know how to recognize and treat them. The removal of the offending drug, addition of symptomatic treatment, and combinations of immunosuppressive therapies can result in significant improvement or resolution of symptoms. Further research is needed to better understand the mechanism driving these adverse reactions of pembrolizumab, including MG and peripheral neuropathy. A stronger understanding of these adverse reactions may allow us to identify risk factors involved in the development of symptoms.
